# Multiple Metabolic Phenotypes as Screening Criteria Are Correlated With the Plant Growth-Promoting Ability of Rhizobacterial Isolates

**DOI:** 10.3389/fmicb.2021.747982

**Published:** 2022-01-05

**Authors:** Peng Shi, Jianli Zhang, Xingyue Li, Liyun Zhou, Hui Luo, Li Wang, Yafan Zhang, Minxia Chou, Gehong Wei

**Affiliations:** State Key Laboratory of Crop Stress Biology in Arid Areas, Shaanxi Key Laboratory of Agricultural and Environmental Microbiology, College of Life Sciences, Northwest A&F University, Yangling, China

**Keywords:** plant growth-promoting rhizobacteria, preliminary screening criteria, metabolic phenotypes, nutrient substrate, root exudates

## Abstract

Efficient screening method is the prerequisite for getting plant growth-promoting (PGP) rhizobacteria (PGPR) which may play an important role in sustainable agriculture from the natural environment. Many current traditional preliminary screening criteria based on knowledge of PGP mechanisms do not always work well due to complex plant–microbe interactions and may lead to the low screening efficiency. More new screening criteria should be evaluated to establish a more effective screening system. However, the studies focused on this issue were not enough, and few new screening criteria had been proposed. The aim of this study was to analyze the correlation between the metabolic phenotypes of rhizobacterial isolates and their PGP ability. The feasibility of using these phenotypes as preliminary screening criteria for PGPR was also evaluated. Twenty-one rhizobacterial isolates were screened for their PGP ability, traditional PGP traits, and multiple metabolic phenotypes that are not directly related to PGP mechanisms, but are possibly related to rhizosphere colonization. Correlations between the PGP traits or metabolic phenotypes and increases in plant agronomic parameters were analyzed to find the indicators that are most closely related to PGP ability. The utilization of 11 nutrient substrates commonly found in root exudates, such as D-salicin, β-methyl-D-glucoside, and D-cellobiose, was significantly positively correlated with the PGP ability of the rhizobacterial isolates. The utilization of one amino acid and two organic acids, namely L-aspartic acid, α-keto-glutaric acid, and formic acid, was negatively correlated with PGP ability. There were no significant correlations between four PGP traits tested in this study and the PGP ability. The ability of rhizobacterial isolates to metabolize nutrient substrates that are identical or similar to root exudate components may act as better criteria than PGP traits for the primary screening of PGPR, because rhizosphere colonization is a prerequisite for PGPR to affect plants.

## Introduction

The rhizosphere, which is the niche influenced by plant roots, is a hot spot for microbial activities in the soil (Hinsinger and Marschner, 2006). Some microorganisms, termed plant growth-promoting rhizobacteria, colonize the rhizosphere and benefit plant growth by producing phytohormones, facilitating plant nutrient acquisition, and antagonizing plant pathogens (Lugtenberg and Kamilova, 2009; Ramos-Solano et al., 2010; Ambrosini et al., 2012; Khan et al., 2019). At present, plant growth-promoting rhizobacteria (PGPR) are regarded as an important component of biofertilizers and have great potential for application in sustainable agriculture (Vessey, 2003; Backer et al., 2018).

In the natural environment, rhizosphere microorganisms interact with plants and regulate their growth via the formation of complex communities (Chaney and Baucomn, 2020). In order to use PGPR as artificial inoculants in practice, it is necessary to isolate pure strains. However, there is a huge number and great diversity of microorganisms that colonize the rhizosphere, and new culture techniques allow us to obtain thousands of isolates simultaneously from the environment. Therefore, it remains challenging to identify a few key strains with the best PGP performance by screening a large number of isolates obtained from the rhizosphere niche (Bai et al., 2015; Ling et al., 2015). In addition, the performance of PGPR may be affected by climatic and environmental conditions, including soil type, temperature, plant cultivar, and interactions with other microorganisms. The data from field trials therefore provide the most accurate basis for screening the best PGPR strains for application in agriculture (Tabassum et al., 2017). Despite this, the large time requirements and high cost of field trials limit their application for large-scale screening of PGPR strains (Moretti et al., 2020).

Many studies have screened for PGPR using a relatively efficient protocol involving the following three steps (Kumar et al., 2015; Soldan et al., 2019; Vasseur-Coronado et al., 2020). The first step is preliminary screening: a small proportion of candidate strains that are most likely to be PGPR are selected, based on the screening of some PGP traits *in vitro*, from hundreds or even thousands of randomly obtained isolates. The second step is evaluation in simulated “real environmental conditions”: some strains exhibiting a PGP ability are screened out by evaluating their effects on plant growth in a greenhouse with sterile substrate or real soil. The last step is field evaluation: several strains with the best PGP ability are identified based on practical application. Because the results of greenhouse and field trials provide direct evidence for the PGP ability of the strains, these data are meaningful for the screening of PGPR strains. However, for the overall screening process, it may be more important to improve the accuracy and efficiency of the *in vitro* preliminary screening, because most of the candidates (usually > 90%) are eliminated in this step (Di Benedetto et al., 2019; Nordstedt and Jones, 2020).

Most PGP traits that are currently used as key indicators in the preliminary screening of PGPR are designed based on known PGP mechanisms, such as solubilizing phosphate and producing siderophores. However, these indicators may not work as well in practice as theoretically predicted due to the variety of PGP mechanisms, the differences between rhizosphere and *in vitro* environments, and the functional redundancy of rhizosphere microorganisms (Finkel et al., 2017; Canto et al., 2020). Many studies have shown a lack of connection between these PGP traits and the actual PGP ability of candidate strains (Backer et al., 2018). For example, Rajendran et al. (2008) screened out three PGPR strains that cannot solubilize phosphates but can produce siderophores. Meanwhile, Zhang et al. (2017) reported three PGPR strains with high phosphate-solubilizing ability that cannot produce siderophores. These phenomena suggest that PGP traits may not be the best criteria for the preliminary screening of PGPR; more universal and effective indicators should be identified. However, at present, many studies still use these traditional PGP traits as the primary screening criteria of PGPR, and few researches have focused on evaluating and proposing novel and easily detectable screening criteria.

As members of the rhizosphere microbiome, PGPR are involved in complex interactions with plants that enable them to colonize close to the root surface (Backer et al., 2018; Lami et al., 2020). There is increasing evidence indicating that the phenotypes of PGPR strains associated with these interactions (such as production of plant polymer-degrading enzymes and biofilm formation) are essential for their adaptation to the rhizosphere niche, and may be markers of PGPR (Shi et al., 2012; Yuan et al., 2015; Walitang et al., 2017; Valetti et al., 2018). Therefore, it may be reasonable to use phenotypes related to plant–microbe interactions as preliminary screening criteria for PGPR, given that successful colonization is the key prerequisite for PGPR to act on plants (Amaya-Gomez et al., 2020).

Root exudates, which are composed of sugars, amino acids, and organic acids, play an active role in the interactions between plants and rhizosphere microorganisms. Root exudates provide rhizosphere microorganisms with nutrient sources and serve as interacting signals (Bais et al., 2006; Badri and Vivanco, 2009; Shi et al., 2012; Yuan et al., 2015; Hassan et al., 2019; Canto et al., 2020). The capacity of rhizosphere microorganisms to utilize the nutrients in root exudates is a vital metabolic feature, both for PGPR and pathogens (Rodriguez et al., 2019; Pascale et al., 2020). Many studies have explored the nutrient utilization capacities of plant pathogens and these capacities have been regarded as an indicator of pathogenicity or for biological control evaluation (Ji and Wilson, 2002; Irikiin et al., 2006; Jian et al., 2019; Zhang et al., 2019; Huang et al., 2020). However, the potential of using nutrient utilization capacities as main screening criteria of PGPR has not been paid enough attention. In particular, the quantitative relationship between the nutrient utilization capacities and PGP ability of strains has been rarely reported, and the possibility of using these nutrient utilization capacities as preliminary screening indicators has hardly been evaluated.

In legumes, the root nodule is a specialized organ that plays a pivotal role in nitrogen supply. In the case of the soybean–rhizobium symbiosis, PGPR can promote both host plant growth (Moretti et al., 2020) and rhizobium symbiotic nodulation (Han et al., 2020). This symbiosis system may be an ideal model to assess the accuracy and universality of PGPR prescreening indicators because it can be affected by microorganisms via more diverse mechanisms than the single plant system. Thus, in the present study, the soybean–rhizobium symbiosis system was used to evaluate the correlations between multiple nutrient utilization phenotypes of rhizobacteria and their PGP ability. Further, the feasibility of using these phenotypes as the main indicators for the pre-screening of PGPR was explored.

## Materials and Methods

### Sampling and Soil Characterization

Using a trowel, several individual plants of the soybean (*Glycine max* [L.] Merrill) cultivar Zhonghuang 13 were uprooted randomly from a field in Yangling, Shaanxi Province, China (34°16′ N, 108°4′ E). The plant sampling was carried out during the flowering stage of soybean in June 2009. Three of the plants with the best growth (each had more than 50 large red nodules) were immediately transported to the laboratory for bacterial isolation. The soil (Lou soil) was also sampled from the same field, and the basic soil characteristics were analyzed at the Test Center of Northwest A&F University (Yangling, China). The soil had a pH of 7.84 and contained a total N content of 980 mg kg^–1^, available N of 10.83 mg kg^–1^, total P of 1 g kg^–1^, available P of 6.48 mg kg^–1^, and available Fe of 9.4 mg kg^–1^.

### Rhizobacterial and Rhizobial Isolation

Rhizobacteria were isolated from the surface of root nodules according to the method described by Kuklinsky-Sobral et al. (2004). Briefly, after the bulk soil was removed, all red nodules were picked off the roots using aseptic forceps. The nodules were washed using sterile water three times to remove the bacterial cells that were not firmly adhered to the nodule surface. Then, the nodules were placed in Erlenmeyer flasks containing glass beads and saline solution (0.7% NaCl). The flasks were shaken at 150 rpm for 1 h at 28°C. After agitation, an appropriate dilution was plated onto nutrient agar plates (ATCC ^®^ Medium 3) and the plates were incubated at 28°C for 2–7 days. Visually different colonies were selected and purified by repeatedly streaking.

After isolating the rhizobacteria, the nodules were immediately further used to isolate rhizobia. The nodules were surface sterilized with 75% (v/v) alcohol for 30 s, followed by 1% (w/v) sodium hypochlorite for 4 min, and then rinsed six times with sterile distilled water. Subsequently, 59 large red nodules (a quarter of the total nodules) were crushed with aseptic forceps and streaked onto yeast mannitol agar plates (Zhao et al., 2010); the plates were incubated at 28°C for 3–15 days. Rhizobial colonies were purified by repeatedly streaking and checked via observation of the colony and cellular morphology and nodulation tests (Zhao et al., 2010). The isolate that was found in the largest number of nodules was selected for co-inoculation experiments.

### Rhizobacterial and Rhizobial Characterization

All rhizobacterial and rhizobial isolates were screened for their PGP traits. Mineral phosphate-solubilizing activity was measured using agar plates with 10.0 g L^–1^ Ca_3_(PO_4_)_2_ according to Qin et al. (2011). Chitinase activity was measured using agar plates with 15.0 g L^–1^ colloidal chitin according to El-Tarabily et al. (2000). Siderophore production was tested using chrome azurol S agar plates according to Bal et al. (2013). All isolates were incubated at 28°C. The ratio of the diameter of the clear zone (halo) or color halo surrounding the colony to the diameter of the colony was used to evaluate the ability of the isolates to solubilize phosphate and produce chitinase and siderophores (Kobayashi et al., 1995; Rau et al., 2009; Kumar et al., 2012; Marra et al., 2012; Suresh, 2012). The indole acetic acid (IAA) production was tested using the method according to Bal et al. (2013). Briefly, the isolates were cultured in King’s B medium with agitation at 150 rpm for 7 days at 28°C. The cultures were centrifuged at 9600 *× g* for 10 min. The supernatant was mixed with Salkowski reagent (1:1 v/v) and placed in darkness for 30 min. The optical density was read at 530 nm on a LAMBDA 35 UV/Vis spectrophotometer (PerkinElmer, Shelton, CT, United States). The IAA concentration was calculated according to the IAA standard curve (0–100 mg L^–1^).

All rhizobacterial and rhizobial isolates were identified by sequencing the 16S rRNA gene. The rhizobacterial and rhizobial isolates were grown in 5 mL of tryptone yeast extract broth (Sharma et al., 2009) and yeast mannitol broth (Zhao et al., 2010), respectively, at 28°C for 2 days with agitation (150 rpm). Then, genomic DNA was extracted with the method described by Zhao et al. (2010) and used as the template. The full-length 16S rRNA gene was amplified using the primers P1 (5′-CGGG ATCCAGAGTTTGATCCTGGTCAGAACGAACGCT-3) and P6 (5′-CGGGATCCTACGGCTACCTTGTTACGACTTCACC CC-3) (Zhao et al., 2010). The PCR products were digested separately using the restriction endonucleases *Hha*I, *Hae*III, and *Hinf*I with the method described by Zhao et al. (2010). The restricted fragments were analyzed using 2% (w/v) agarose gel electrophoresis. Isolates sharing identical restriction fragment length polymorphism patterns were defined as the same 16S rRNA genotype. The 16S rRNA genes of representative isolates with different 16S rRNA genotypes were sequenced and aligned using the EzBioCloud Database^1^.

Based on the results of the PGP traits screening and 16S rRNA gene sequence alignment, 21 rhizobacterial isolates were selected for a single inoculation experiment to examine the effects of them on soybean plants without the rhizobial symbiont. Seeds of soybean cultivar Zhonghuang 13 were sorted for uniformity, and were surface sterilized with 75% (v/v) alcohol and 1% (w/v) sodium hypochlorite. The seeds were then germinated on 1.2% (w/v) water agar plates at room temperature in the dark for 3 days (Fox et al., 2011). The seedlings were then planted in pots filled with a sterilized vermiculite–perlite mixture (2:1, v/v). The rhizobacterial isolates were cultured in tryptone yeast extract broth (Sharma et al., 2009) and prepared in cell suspensions (optical density of 0.55 at 600 nm, approximately 10^8^–10^9^ cells mL^–1^). The cell suspensions were injected into the rhizosphere of the soybean plants when the cotyledons had unfolded. The plants were cultivated in a greenhouse and harvested at 30 days post-inoculation (dpi) to measure the dry weights of the roots and shoots and the number of root nodules. The single inoculation experiment was repeated three times, as described in co-inoculation experiments. The ratio of the dry weight of the inoculated plants (nine replicates) to that of the non-inoculated plants (24 replicates) was used to assess the effects of the isolate on the plant.

Metabolic phenotype analysis was carried out for the 21 rhizobacterial isolates using the Biolog™ Gen III ID system (Biolog, Hayward, CA, United States) according to protocol A provided by the manufacturer. Briefly, isolates were incubated on nutrient agar plates (ATCC ^®^ Medium 3) for 24 h at 28°C. All isolates (two replicates per isolate) were then inoculated into inoculation fluid A (IF-A, Biolog, Hayward, CA, United States) to give an optical density of 95%. Of the cell suspensions, 100 μL was inoculated into each well of a GEN III microplate. The plates were incubated at 28°C, and absorbance data were recorded at 12, 24, and 36 h using the Biolog station ELx808BLG (Biolog, Hayward, CA, United States) (Van Assche et al., 2017). The data collected at 36 h were used in the subsequent analysis because the readings of the two replicates were most consistent (Wozniak et al., 2019). The Well Color Development (WCD; end-point absorbance value of each well – the negative control well value; set to zero if the value was a small negative number) value was used to evaluate the ability of an isolate to utilize nutrient substrates (two replicates for one nutrient substrate).

### Co-inoculation Experiments

To verify whether the effects of the rhizobacterial isolates on the soybean–rhizobium symbiosis were stable, co-inoculation experiments were performed three times over two different years (2012 and 2014). In the first experiment, which was conducted in 2012, the 21 isolates were randomly divided into six groups: (1) CCNWSP2, CCNWSP26, and CCNWSP31; (2) CCNWSP33, CCNWSP68, CCNWSP76, and CCNWSP78; (3) CCNWSP27, CCNWSP30, and CCNWSP60; (4) CCNWSP11, CCNWSP13-2, CCNWSP25, and CCNWSP46; (5) CCNWSP10, CCNWSP15, and CCNWSP21; and (6) CCNWSP4, CCNWSP13-4, CCNWSP21-1, and CCNWSP92. The co-inoculation treatment involved CCNWSX1528 and a rhizobacterial isolate, whereas the control treatment involved the rhizobial isolate CCNWSX1528 alone. For each treatment, three replicates (five plants per replicate) were included. The second and third experiments were performed in 2014 by two different researchers at the same time. The treatments were the same as those used for the experiment conducted in 2012, with three replicates (three plants per replicate) per treatment.

During each experiment, seeds of soybean cultivar Zhonghuang 13 were surface sterilized and germinated as performed in the inoculation experiment (Fox et al., 2011). The seedlings were then planted in 1 L pots (one seedling per pot) filled with a sterilized vermiculite–perlite mixture (2:1, v/v). The seedlings were watered with 150 mL of nitrogen-free plant nutrient solution containing the following ingredients: 0.5 g L^–1^ K_2_HPO_4_, 2.0 g L^–1^ Ca_3_(PO_4_)_2_, 0.2 g L^–1^ MgSO_4_⋅7H_2_O, 0.1 g L^–1^ NaCl, and 0.01 g L^–1^ FeCl_3_ (Zhao et al., 2010). All pots were placed in a greenhouse under a 14/10 h light/dark cycle and 25/20°C day/night temperatures.

The rhizobial isolate, CCNWSX1528, was cultured in yeast mannitol broth (Zhao et al., 2010) for 3 days, and the rhizobacterial isolates were cultured in tryptone yeast extract broth (Sharma et al., 2009) for 2 days. Both types of isolate were cultured at 28°C with agitation (150 rpm). Cultures were centrifuged at 1600 *× g* for 10 min, and the cell pellets were suspended in NaCl solution (0.7% w/v) to an optical density of 0.55 at 600 nm (approximately 10^8^–10^9^ cells mL^–1^). When the soybean cotyledons had unfolded, 150 mL of nitrogen-free plant nutrient solution and one of the following inoculants were injected into the rhizosphere: (1) 1 mL of 0.7% NaCl solution and 1 mL of CCNWSX1528 suspension for the control treatment; and (2) 1 mL of rhizobacterial suspension and 1 mL of CCNWSX1528 suspension for the co-inoculation treatment. The plants were cultivated in a greenhouse, watered with 150 mL of nitrogen-free plant nutrient solution every 10 days, and harvested at 30 dpi.

All five (first experiment) or three (second and third experiments) plants in each replicate were pooled to measure the growth and symbiotic nodulation of the plants. The roots, shoots, and root nodules were dried for 4 days at 80°C to measure the root dry weight (RDW), shoot dry weight (SDW), and dry weight of total nodules per plant. The total number of nodules (TNN) and the number of red nodules (RNN) per plant were recorded (Fox et al., 2011; Egamberdieva et al., 2013). The dry weight per nodule (NDW) was calculated as the ratio of the dry weight of total nodules per plant to TNN. The root total nitrogen (RN) and shoot total nitrogen (SN) per plant were analyzed using an automatic Kjeldahl nitrogen analyzer (KJEL-AUTO VS-KT-P; MRK, Tokyo, Japan).

### Statistical Analysis

To facilitate data analysis, the seven plant agronomic parameters were transformed using the equation $R\,a\,t\,i\,o=D\,a\,t\,a_{E\,R}\,/\,D\,a\,t\,a_{\bar{C\,K}}$, where **Data*_*ER*_* denotes the data of each replicate in the co-inoculation treatments, and $D\,a\,t\,a_{\bar{C\,K}}$ denotes the mean of the data in the control treatment. The mean *Ratio* value of the seven parameters was calculated as a comprehensive index (CI) and used to indicate the total effects of the rhizobacterial isolates on the soybean–rhizobium symbiosis system. Forest plot was used to show the *Ratio* of the agronomic parameters of plants co-inoculated with 21 rhizobacterial strains and *Sinorhizobium* sp. CCNWSX1528 to those inoculated with *Sinorhizobium* sp. CCNWSX1528 alone with R package(forestplot). The significance of differences between treatments was determined via one-way analysis of variance (ANOVA) and Dunnett’s tests. Spearman’s correlation coefficients were used to assess the relationships between multiple phenotypes of rhizobacterial isolates and their PGP ability. All statistical analyses were carried out using IBM SPSS Statistics 22 (IBM Corp., Armonk, NY, United States).

## Results

### Isolation and Characterization of Rhizobia and Rhizobacteria

A total of 90 rhizobacterial isolates were obtained from the surface of the soybean nodules and they were screened for traditional PGP traits. The 90 isolates belonged to 13 genera within four phyla: *Microbacterium* (1 isolate) in Actinobacteria; *Sphingobacterium* (5 isolates) in Bacteroidetes; *Bacillus* (24 isolates) and *Lysinibacillus* (9 isolates) in Firmicutes; and *Advenella* (2 isolates), *Agrobacterium* (3 isolates), *Alcaligenes* (17 isolates), *Brevundimonas* (1 isolate), *Paracoccus* (2 isolates), *Pseudochrobactrum* (16 isolates), *Shinella* (1 isolate), *Ensifer* (5 isolates), and *Stenotrophomonas* (4 isolates) in Proteobacteria. Most of these isolates had at least one PGP trait, with the exception of CCNWSP10, CCNWSP24-1, CCNWSP36-1, CCNWSP73-2, CCNWSP75, and CCNWSP81-1. Seven isolates could solubilize mineral phosphate; 39 isolates produced siderophores; 27 isolates showed chitinase activity; and 65 isolates produced IAA (detailed data are shown in Supplementary Table 1).

A total of 126 rhizobial isolates were recovered from the soybean nodules. One of these, namely CCNWSX1528, showed a strong nodulation ability in the nodulation test (28 nodules per plant on average, *n* = 3), and was therefore chosen for plant co-inoculation experiments. The 16S rRNA gene sequence of this isolate (accession number KF735789) showed 99.32 and 99.13% similarity with the sequences of *Ensifer shofinae* CCBAU 251167^T^ and *Ensifer fredii* NBRC 14780^T^, respectively. The four traits of rhizobial isolates CCNWSX1528 were also tested. Its indole acetic acid production was 51.36 ± 4.13 mg L^–1^ (mean ± standard errors, *n* = 3), the ratio of mineral phosphate solubilization halo diameter to colony diameter was 2.10 ± 0.17 (mean ± standard errors, *n* = 3), and it could not produce siderophores and chitinase (Table 1). In the three co-inoculation experiments, soybean plants inoculated with isolates CCNWSX1528 had an average nodule number of 35 ± 2 (mean ± standard errors, *n* = 24), and significantly increased root and shoot dry weight by 29.9 and 46.5% (*p* < 0.01, *n* = 24) compared to the plants no-inoculated, respectively (detailed data are shown in Supplementary Tables 2, 3).

**TABLE 1:** The PGP traits and closest relatives of the rhizobial and rhizobacterial isolates used for the inoculation experiments.

Phylum	Genus	Isolates	Mineral phosphate solubilization^a^	Siderophore production^a^	Chitinase production^a^	Indole acetic acid production (mg L^–1^)	Strain preservation number	GenBank accession number of 16S rDNA sequence	Closest relatives (Sequence similarity)
Proteobacteria	*Ensifer*	CCNWSX1528	2.10 ± 0.17	–^b^	–	51.36 ± 4.13	ACCC19832	KF735789	*Ensifer* (*Sinorhizobium*) *shofinae* CCBAU 251167^T^ (99.32%)
Actinobacteria	*Microbacterium*	CCNWSP60	–	–	–	13.27 ± 0.23	ACCC19329	KF735798	*Microbacterium shaanxiense* CCNWSP60^T^ (100.00%)
Bacteroidetes	*Sphingobacterium*	CCNWSP31	–	1.41 ± 0.08	–	–	ACCC19831	KF735791	*Sphingobacterium yanglingense* CCNWSP36-1^T^ (100.00%)
Firmicutes	*Bacillus*	CCNWSP2	–	–	1.39 ± 0.02	–	ACCC19813	KF735790	*Bacillus proteolyticus* TD42^T^ (99.93%)
		CCNWSP11	–	–	1.37 ± 0.06	14.47 ± 0.35	ACCC19814	KF735802	*Bacillus cereus* ATCC 14579^T^ (98.83%)
		CCNWSP46	–	–	1.43 ± 0.09	14.73 ± 0.84	ACCC19811	KF735801	*Bacillus proteolyticus* TD42^T^ (99.10%)
		CCNWSP76	–	–	1.14 ± 0.03	–	ACCC19812	KF735793	*Bacillus proteolyticus* TD42^T^ (99.10%)
	*Lysinibacillus*	CCNWSP21	–	–	–	33.83 ± 2.83	ACCC19816	KF735803	*Lysinibacillus fusiformis* NBRC 15717^T^ (100.00%)
Proteobacteria	*Advenella*	CCNWSP33	2.66 ± 0.32	2.78 ± 0.28	–	16.93 ± 0.90	ACCC19825	KF735794	*Advenella kashmirensis* subsp. *methylica* PK1^T^ (100.00%)
	*Agrobacterium*	CCNWSP26	–	–	–	22.13 ± 0.64	ACCC19822	KF735792	*Agrobacterium deltaense* YIC 4121^T^ (100.00%)
	*Alcaligenes*	CCNWSP13-2	–	1.28 ± 0.01	–	–	ACCC19828	KF735810	*Alcaligenes faecalis* subsp. *phenolicus* DSM 16503^T^(99.84%)
		CCNWSP13-4	–	1.55 ± 0.07	–	–	ACCC19827	KF735809	*Alcaligenes faecalis* subsp. *phenolicus* DSM 16503^T^(99.22%)
		CCNWSP30	–	1.87 ± 0.07	–	–	ACCC19829	KF735799	*Alcaligenes faecalis* subsp. *phenolicus* DSM 16503^T^ (99.43%)
		CCNWSP78	–	1.97 ± 0.17	–	–	ACCC19826	KF735797	*Alcaligenes faecalis* subsp. *phenolicus* DSM 16503^T^ (100.00%)
	*Brevundimonas*	CCNWSP10	–	–	–	–	ACCC19824	KF735808	*Brevundimonas bullata* IAM 13153^T^ (99.48%)
	*Paracoccus*	CCNWSP27	–	–	–	58.90 ± 6.33	ACCC19823	KF735800	*Paracoccus litorisediminis* GHD-05^T^ (100.00%)
	*Pseudochrobactrum*	CCNWSP4	–	1.42 ± 0.08	–	69.60 ± 6.67	ACCC19820	KF735806	*Pseudochrobactrum asaccharolyticum* DSM 25619^T^ (99.14%)
		CCNWSP21-1	–	1.50 ± 0.03	–	391.5 ± 7.8	ACCC19819	KF735805	*Pseudochrobactrum asaccharolyticum* DSM 25619^T^ (99.06%)
		CCNWSP25	–	–	1.70 ± 0.23	28.93 ± 7.20	ACCC19818	KF735804	*Pseudochrobactrum asaccharolyticum* DSM 25619^T^ (99.20%)
		CCNWSP68	–	1.97 ± 0.28	–	345.2 ± 31.3	ACCC19817	KF735796	*Pseudochrobactrum asaccharolyticum* DSM 25619^T^ (98.78%)
	*Shinella*	CCNWSP92	–	–	–	45.33 ± 11.71	ACCC19821	KF735807	*Shinella zoogloeoides* ATCC 19623^T^ (99.26%)
	*Stenotrophomonas*	CCNWSP15	–	2.22 ± 0.11	2.38 ± 0.22	56.20 ± 6.03	ACCC19830	KF735811	*Stenotrophomonas maltophilia* MTCC 434^T^ (99.65%)

*^a^The ratio of the halo diameter to the colony diameter was used to indicate the ability of an isolate to solubilize mineral phosphate and produce siderophores and chitinase. Data are presented as means ± standard errors (n = 3).*

*^b^– Negative for the trait.*

**FIGURE 1:**
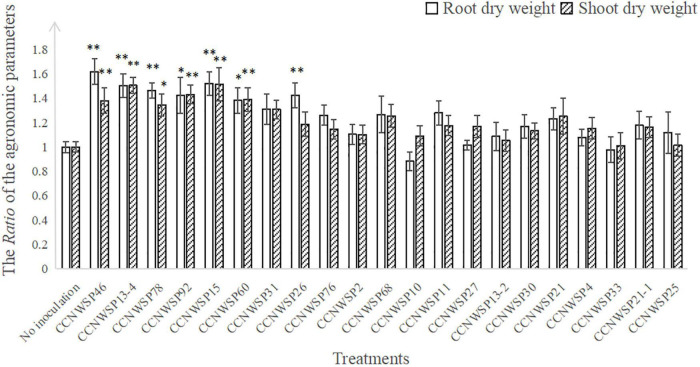
The *Ratio* of the agronomic parameters of plants inoculated with 21 rhizobacterial strains to non-inoculated plants. Data are means of $R\,a\,t\,i\,o=D\,a\,t\,a_{E\,R}/D\,a\,t\,a_{\bar{C\,K}}$ (**Data*_*ER*_* denotes the data of each replicate in the rhizobacteria inoculation treatments, and $D\,a\,t\,a_{\bar{C\,K}}$ denotes the mean of the data in the non-inoculation treatment). There are nine replicates for rhizobacteria inoculation treatments, 24 replicates for non-inoculation treatment. Bars represent standard errors. * And ^**^ are indicating significant differences (*p* < 0.05 and *p* < 0.01, respectively) among treatments (inoculation vs. non-inoculation) according to Dunnett’s test.

Among 12 of the 13 rhizobacterial genera (all excluding the rhizobial genus *Ensifer*), 21 isolates were selected for the subsequent experiments (Table 1). In the single inoculation experiment without the rhizobium, seven isolates, namely CCNWSP46, CCNWSP13-4, CCNWSP78, CCNWSP92, CCNWSP15, CCNWSP60, and CCNWSP26, significantly promoted plant growth in terms of increasing root dry weight (by 38–62%) and shoot dry weight (by 34–51%). None of these 21 isolates formed nodules with soybean roots (Figure 1 and detailed data are shown in Supplementary Tables 2, 4).

**FIGURE 2:**
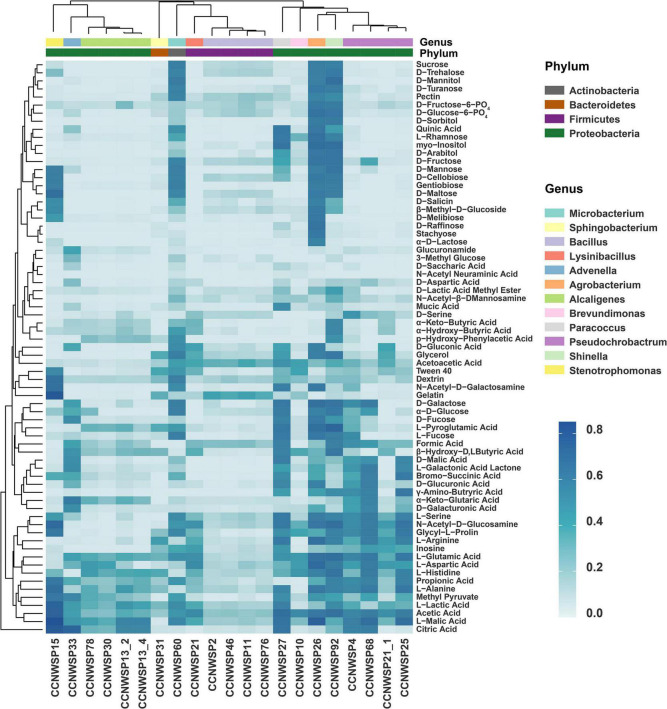
Heat map shows the Well Color Development values of 21 rhizobacterial isolates, representing their ability to metabolize 71 carbon substrates in Biolog Gen III microplates. Data are the mean values of two replicates in different microplates.

All of the 21 rhizobacterial isolates were screened for their ability to metabolize 71 nutrient substrates (Figure 2 and detailed data are shown in Supplementary Table 5). It was found that these metabolic phenotypes were genus specific. The isolates belonging to the same genus had similar metabolic phenotypes with respect to the utilization of various nutrient substrates. Meanwhile, the isolates of different genera showed diverse metabolic patterns. Some of the isolates (CCNWSP92, CCNWSP15, CCNWSP60, and CCNWSP26), which exhibited better PGP effects in the single inoculation experiment, showed stronger ability to utilize multiple sugar substrates, such as D-Maltose, D-trehalose, D-cellobiose, and gentiobiose. However, other isolates (CCNWSP13-4 and CCNWSP78) showed a different pattern of nutrient substrate metabolism; they could not use those more available sugar substrates, but were able to utilize *p*-hydroxyphenylacetic acid, which is a carbon source that is relatively harder to be used.

### Effects of Rhizobacterial Isolates on the Soybean–Rhizobium Symbiosis System

**FIGURE 3:**
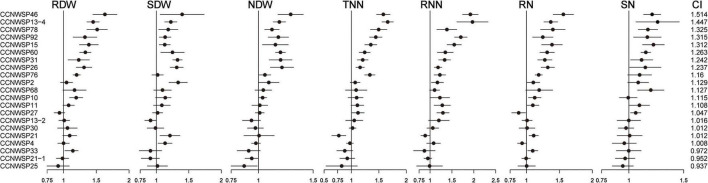
The *Ratio* of the agronomic parameters of plants co-inoculated with 21 rhizobacterial strains and *Sinorhizobium* sp. CCNWSX1528 to those inoculated with *Sinorhizobium* sp. CCNWSX1528 alone. The dark dots represent means of $R\,a\,t\,i\,o=D\,a\,t\,a_{E\,R}/D\,a\,t\,a_{\bar{C\,K}}$ (**Data*_*ER*_* denotes the data of each replicate in the co-inoculation treatments, and $D\,a\,t\,a_{\bar{C\,K}}$ denotes the mean of the data in the single inoculation treatment). There are nine replicates for co-inoculation treatments. Bars represent 95% confidence intervals. RDW, root dry weight; SDW, shoot dry weight; NDW, dry weight per nodule; TNN, total number of nodules; RNN, number of red nodules; RN, root total nitrogen; SN, shoot total nitrogen; and CI, comprehensive index.

The 21 rhizobacterial isolates were tested in the co-inoculation experiments. Of these, 13 isolates showed capacity to markedly increase at least one plant agronomic parameter (Figure 3 and detailed data are shown in Supplementary Tables 3, 6). When co-inoculated with the rhizobium CCNWSX1528, 10 isolates significantly increased the RDW by 19–63%, 7 isolates increased the SDW by 16–30%, 6 isolates increased the NDW by 22–40%, 8 isolates increased the TNN by 21–66%, 8 isolates increased the RNN by 30–97%, 10 isolates increased the RN by 19–56%, and 6 isolates increased the SN by 17–26%. These 13 isolates increased the CI by 5–51%.

The 13 isolates belong to nine genera, namely *Bacillus* (four isolates), *Alcaligenes* (two isolates), *Stenotrophomonas* (one isolate), *Microbacterium* (one isolate), *Sphingobacterium* (one isolate), *Agrobacterium* (one isolate), *Pseudochrobactrum* (one isolate), *Brevundimonas* (one isolate), and *Paracoccus* (one isolate). There were only three genera in which the isolates did not show significant PGP ability. Eight isolates (CCNWSP46, CCNWSP13-4, CCNWSP78, CCNWSP92, CCNWSP15, CCNWSP60, CCNWSP31, and CCNWSP26) showed better PGP effects with respect to almost all parameters and had the highest CI values (1.237–1.514). Meanwhile, the CI values of the other isolates were less than 1.2. Among the eight isolates with the best PGP effects, only CCNWSP31 did not significantly promote plant growth in the single inoculation experiment without rhizobium.

### The Correlations Between Rhizobacterial Phenotypes and Plant Growth-Promoting Ability

**FIGURE 4:**
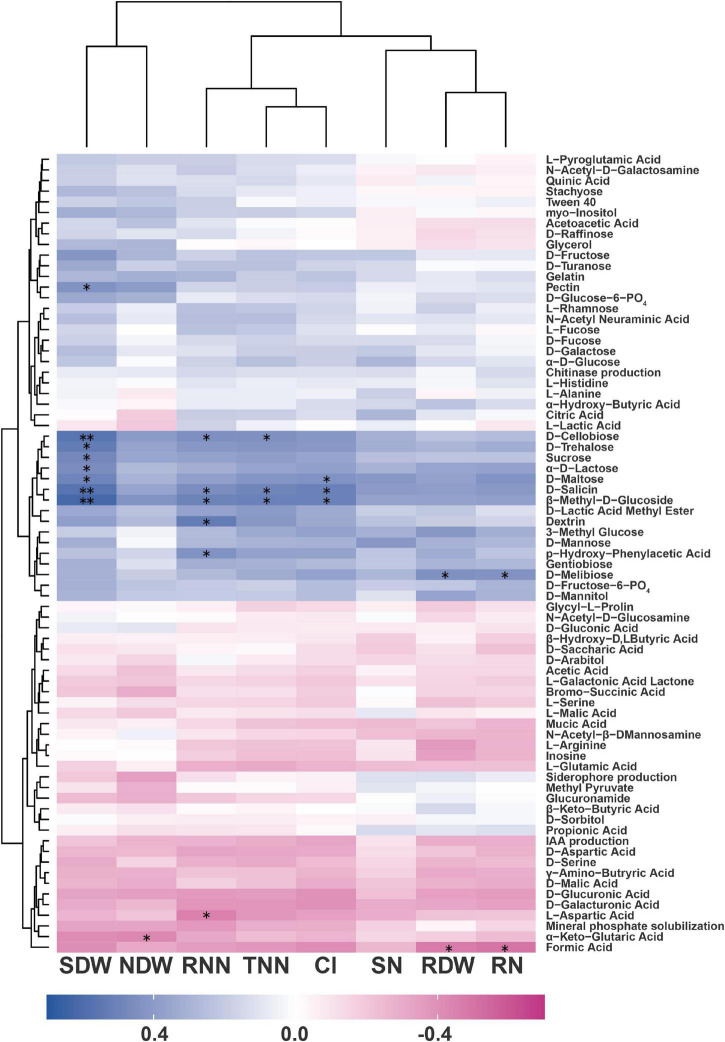
Heat map shows the Spearman’s correlation coefficients between the metabolic phenotypes of the rhizobacterial isolates and their promotional effects on the soybean–rhizobium system. RDW, root dry weight; SDW, shoot dry weight; NDW, dry weight per nodule; TNN, total number of nodules; RNN, number of red nodules; RN, root total nitrogen; SN, shoot total nitrogen; and CI, comprehensive index. *, Significant correlation at *p* < 0.05 and ^**^, significant correlation at *p* < 0.01 (*n* = 21).

Spearman’s correlation analysis was carried out to explore the potential relationships between the eight soybean plant parameters (RDW, SDW, NDW, TNN, RNN, RN, SN, and CI) and the multiple phenotypes of the rhizobacterial isolates, including four PGP traits (solubilizing phosphate and producing IAA, chitinase, and siderophores) and 71 metabolic phenotypes (WCD, Biolog™ Gen III). The improvement of plant parameters was significantly correlated with the metabolism of some substrates, but not with any PGP traits (Figure 4 and detailed data are shown in Supplementary Table 7).

The metabolism of 11 substrates was significantly positively related to PGP ability. Specifically, D-melibiose utilization was related to an increase in RDW (*r* = 0.443). D-Maltose, D-trehalose, D-cellobiose, sucrose, α-D-lactose, β-methyl-D-glucoside, D-salicin, and pectin utilization were related to increases in SDW (*r* = 0.435–0.621). D-Cellobiose, β-methyl-D-glucoside, and D-salicin utilization were related to increases in TNN (*r* = 0.443–0.521). Dextrin, D-cellobiose, β-methyl-D-glucoside, D-salicin, and *p*-hydroxyphenylacetic acid utilization were related to increases in RNN (*r* = 0.435–0.530). D-Melibiose utilization was related to an increase in RN (*r* = 0.437). D-Maltose, β-methyl-D-glucoside, and D-salicin utilization were related to increases in CI (*r* = 0.435–0.510). In addition, the metabolism of three substrates was significantly negatively related to PGP ability. They are, L-aspartic acid utilization was related to RNN (*r* = −0.453), α-keto-glutaric acid utilization was related to NDW (*r* = −0.433), and formic acid utilization was related to RDW (*r* = −0.475) and RN (*r* = −0.499).

## Discussion

### Selection of Test Isolates, Plant Agronomic Parameters, and Plant Growth-Promoting Traits

This study was carried out to explore the possibility of using multiple metabolic phenotypes as criteria for the preliminary screening of PGPR. The aim of the study was achieved by evaluating the correlations between the metabolic phenotypes of PGPR isolates and their PGP ability, and between the PGP traits of the isolates and their PGP ability. In order to obtain more representative criteria, focus was placed on the selection of test isolates, plant agronomic parameters, and PGP traits when designing the experiment.

Among the 90 rhizobacterial isolates obtained in this study, Proteobacteria (51 isolates) was the most dominant phylum, followed by Firmicutes (33 isolates). In addition, Bacteroidetes (five isolates) and Actinobacteria (one isolate) were identified. There is a general assumption that fast-growing, easily cultivable Proteobacteria are the dominant rhizosphere colonizers (Kristen et al., 2009). Xu et al. (2009) reported that Proteobacteria, Actinobacteria, Bacteroidetes, Nitrospirae, Firmicutes, Verrucomicrobia, and Acidobacteria could colonize the soybean rhizosphere, with Proteobacteria being the dominant group (>40% of the bacterial community). Zhang et al. (2018) also found that Proteobacteria, Firmicutes, and Actinobacteria were the prominent phyla in the rhizosphere microbial communities of soybean at 50 sites across China. The results of the present study are thus consistent with these previous findings.

At the genus level, the dominant rhizobacteria isolated in the present study were *Bacillus* (24 isolates), *Alcaligenes* (17 isolates), *Pseudochrobactrum* (16 isolates). Besides *Ensifer*, which is a common symbiotic rhizobial genus, some of the other genera isolated in this study have also been previously identified as potential sources of PGPR (Vessey, 2003; Khan et al., 2017; Rahmoune et al., 2017). Here, 21 isolates were selected from the 90 rhizobacterial isolates to represent the 12 genera (all except *Ensifer*) as follows: four isolates were selected from each of the three genera containing the largest number of isolates (*Bacillus*, *Alcaligenes*, and *Pseudochrobactrum*), and one isolate was selected from each of the remaining nine genera (*Microbacterium*, *Sphingobacterium*, *Lysinibacillus*, *Advenella*, *Agrobacterium*, *Brevundimonas*, *Paracoccus*, *Shinella*, and *Stenotrophomonas*). The co-inoculation results showed that PGPR isolates accounted for a large proportion of both the genera (9/12) and isolates (13/21). In accordance with the results of previous studies (Vessey, 2003; Khan et al., 2017; Rahmoune et al., 2017; Backer et al., 2018), the rhizobacterial isolates in the present study that belonged to *Bacillus*, *Alcaligenes*, *Stenotrophomonas*, *Microbacterium*, *Sphingobacterium*, and *Agrobacterium* were associated with significant PGP effects. This indicates that there are a large number of PGPR inhabiting the soybean rhizosphere, and they are widely distributed across several different genera.

In this study, seven plant agronomic parameters (such as RDW, SDW, and NDW) and one index (CI) were used to describe the PGP effects of rhizobacterial isolates. PGPR can affect plants through various and complex pathways (Backer et al., 2018; Khan et al., 2019; Rodriguez et al., 2019). The diversity of PGP mechanisms means that multiple plant agronomic parameters may change due to the action of PGPR. So, the standards for evaluating the performance of PGPR should include not only plant growth traits, such as shoot elongation, plant height, plant biomass, and yield (Tabassum et al., 2017; Rosier et al., 2021), but also more targeted indicators, such as root architecture, flowering time, and plant enzyme activity, in some specific cases (Chaney and Baucomn, 2020; Chu et al., 2020; Lee et al., 2020; Mahdavi et al., 2020). In addition, a variety of comprehensive indexes calculated based on several plant parameters have previously been used as the main standard to evaluate the quality of fruit, plant vigor, and physiological parameters. These comprehensive indexes are considered more representative than single parameters (Wang et al., 2011; Guo et al., 2019; Dey and Raghuwanshi, 2020).

In the present study, a soybean–rhizobium symbiosis system was used to evaluate the PGP effects of rhizobacterial isolates on plants. Comparing the plant agronomic parameters under the two experimental conditions (with and without the rhizobium), it was found that seven isolates that showed PGP effects in the single inoculation experiment, could also promote the symbiosis in co-inoculation experiments. Interestingly, six isolates that did not show PGP effects in single-inoculation experiments, increased at least one plant agronomic parameter in co-inoculation experiments. This phenomenon suggests that, in addition to directly regulating plant growth in the soybean–rhizobium symbiosis system, the PGPR isolates could act via other PGP mechanisms, such as by facilitating symbiotic nodulation. Therefore, seven plant agronomic parameters, including two related to plant growth (RDW and SDW), three related to root nodulation (TNN, RNN, and NDW), and two related to nitrogen fixation (RN and SN), were selected and integrated into one index (CI) to assess the effects of PGPR on plant growth and symbiotic nodulation through multiple mechanisms.

Four traditional PGP traits were selected to evaluate their reliability as pre-screening criteria for PGPR in the present study. Two of the traits, mineral phosphorus solubilization and siderophore production, are associated with nutrient availability. Mineral phosphorus solubilization can increase soil phosphorus availability in the environment where available phosphorus is scarce and mineral phosphorus is abundant (Brito et al., 2020). Siderophore production can convert the insoluble ferric iron (Fe^3+^) into the soluble ferrous form (Fe^2+^) and increase the absorption of iron by plants, or can control plant pathogens by depriving them of iron in an iron-deficient environment (Gopalakrishnan et al., 2015; Ghazy and El-Nahrawy, 2020). In order to create conditions for these two traits to function, Ca_3_(PO_4_)_2_ and FeCl_3_ were added to the sterile substrate as the main phosphorus and iron sources, respectively. The two further traits tested in this study were IAA and chitinase production. IAA is one of the most important auxins produced by PGPR and thus IAA production can be directly associated with plant growth (Spaepen and Vanderleyden, 2011). Meanwhile, chitinase production may affect nodulation by hydrolyzing the nod factor, which is an important signaling molecule involved in nodulation (Jung et al., 2008).

Many studies have used these four PGP traits described above as the main markers of PGPR (Li et al., 2020; Toscano-Verduzco et al., 2020). However, in the present study, no significant correlations were found between these four PGP traits and the actual PGP ability of the rhizobacterial isolates. Moreover, one isolate (CCNWSP10), which did not display any of these four PGP traits, showed PGP effects in the inoculation experiments. The reliability of using PGP traits as the primary basis for the preliminary screening of PGPR in the laboratory has also been questioned by other researchers. These questions have arisen because the action of these PGP mechanisms is dependent on the environment and there are many other unknown PGP mechanisms (Finkel et al., 2017; Backer et al., 2018). Therefore, instead of the traditional PGP traits, other traits which have stronger correlations with the actual PGP ability should be identified and used as indicators for primary PGPR screening (Amaya-Gomez et al., 2020).

### Feasibility of Using Metabolic Phenotypes as Screening Indicators

The metabolic phenotypes of microorganisms, with respect to the utilization of nutrient substrates found in root exudates, are closely related to the ability of the microorganisms to colonize the rhizosphere of plants. This colonization is a necessary prerequisite for plant–microbe interactions (Zhalnina et al., 2018; Hassan et al., 2019). Amaya-Gomez et al. (2020) suggested that colonization capacity can be used as an indicator for the evaluation of PGPR. It can be proposed that the capacity of PGPR to utilize nutrient substrates may be a more universal and stable predictor of their possible effects on plants. Therefore, in the present study, rhizobacterial isolates were screened for their ability to utilize multiple nutrient substrates. It was found that 11 of the nutrient utilization phenotypes were significantly positively correlated with increases in specific plant agronomic traits.

Most of these substrates are common components of root exudates (Prasanna et al., 2010; Zhang et al., 2020). Some of them, such as dextrin, lactose, D-melibiose and *p*-hydroxyphenylacetic acid, are mainly utilized as nutrients by rhizobacteria (Prasanna et al., 2010; Oksinska et al., 2011; Silini-Cherif et al., 2012). Dextrin is an available carbon source for PGPR (Silini-Cherif et al., 2012). Lactose is one of the main carbon sources impacting the functional diversity of the soybean rhizosphere (Zhang et al., 2020). Some PGPR strains also show the ability to utilize it (Kumar et al., 2016). Melibiose is a degradation product of raffinose which is widely present in plant seeds and can be released to rhizosphere (Bringhurst et al., 2001; Andersen et al., 2005; Meyer et al., 2018). Some PGPR isolates have positive performance in term of utilizing melibiose (Prasanna et al., 2010). The present study not only showed once again that these carbon sources could be utilized by rhizobacteria, but also showed that there were significant correlations between the phenotypes of using these carbon sources and PGP ability. Many previous studies have suggested that the ability of strains to utilize specific carbon sources helps them colonize the rhizosphere. For example, Kragelund and Nybroe (1996) reported that 2,4-dichlorophenoxyacetic acid was the main carbon source for an *Alcaligenes* strain during rhizosphere colonization. Oksinska et al. (2011) suggested that *p*-hydroxyphenylacetic acid could influence colonization efficacy of PGPR. Meyer et al. (2018) showed that the assimilation of melibiose was crucial for plant pathogens to gain an advantage in the rhizosphere. Based on these reports, we suggested that the correlations between the phenotypes and PGP ability could be attributed to that utilizing these carbon sources could support the PGPR strains more nutrients, help them gain some benefits in rhizosphere colonization and growth, and play the PGP roles better.

In addition to serving as nutrients, some of these substrates, such as sucrose, maltose and pectin, can further influence the ability of strains to form biofilms and express PGP mechanisms in the environment (Patel et al., 2008; Al-Ali et al., 2018). Sucrose and maltose exist in the root exudates of many plants and are two of the main nutrients used by rhizosphere microorganisms (Carvalhais et al., 2011; Rameshkumar et al., 2016). As carbon sources, the utilization of sucrose and maltose by bacteria contributes to the formation of biofilm which is very important for colonization (Al-Ali et al., 2018). In addition, the study of Taghavi et al. (2010) showed that the sucrose metabolism could affect the synthesis of plant growth promoting phytohormones by the plant endophytic bacterium. Carvalhais et al. (2011) suggested that the limitation of nutrient supply might lead to more release of maltose. More sucrose and maltose may improve strain’s ability to dissolve phosphorus in the environment (Mukhtar et al., 2017). Pectin is a component in the plant cell wall (Chudzik et al., 2018). It also can be found in secretions of rhizosphere and utilized by rhizobacteria (Li et al., 2012). Wu et al. (2015) reported that pectin could serve as an environmental factor in the stimulation of the biofilm formation, increased production of secondary metabolites by PGP strain and improved its colonization density. Mise et al. (2020) suggested that the actual phosphorus solubilization ability of the phosphate-solubilizing bacteria strains in soil was related to their pectin-degrading activity. Some studies, including ours, showed the lack of correlations between the PGP traits as main pre-screening indicators and the actual PGP ability of strains, and attributed it to the fact that the PGPR strains could not adapt to the rhizosphere environment, and/or could not express its PGP traits in the rhizosphere just like *in vitro* (Finkel et al., 2017; Backer et al., 2018). Based on the present study, it could be suggested that some metabolic phenotypes which could help the strains adapt to the rhizosphere environment and express the PGP ability in this environment might be more suitable for predicting the PGP effects of bacterial strains than the PGP traits tested *in vitro*.

Some of these substrates, such as trehalose and salicin, may participate in the immune response of plants in addition to being microbial carbon sources. D-trehalose showed relatively strong correlation (*r* > 0.5) with plant growth (SDW). The result was consistent with the study of Radha and Rao (2014) which tested seven PGP strains and found all of them could utilize trehalose. Trehalose is widely present in bacteria, fungi, insects, plants and other organisms, and associated with a variety of physiological functions (Wang et al., 2020). It can be accumulated in nodules and do help to rhizobial symbiont when legumes form symbioses with rhizobia (Salminen and Streeter, 1986; Garg and Singla, 2016). Barraza’s study showed that the trehalose biosynthesis-related genes of legumes played a key role in regulating the content of trehalose in root nodules and impacted on plant growth and nodule formation (Barraza et al., 2016). All of the strains used in our study were isolated from nodule surface which might have higher trehalose content than other rhizosphere niches. We proposed that the ability to decompose and utilize trehalose might be conducive to the colonization and activity maintenance of the strains in this area. Furthermore, the trehalose is a signal of microbial pathogen attack, and can trigger plant defense responses (Lunn et al., 2014). There is always a large amount of trehalose accumulated in the infected organs as they infected by pathogen (Brodmann et al., 2002). The excessive accumulation of trehalose in plants may have negative effects on plants (Goddijn et al., 1997; Shim et al., 2019). Gravot et al. (2011) found that AtTRE1 gene in roots and hypocotyls of *Arabidopsis thaliana* was induced when the infection of pathogen led to the accumulation of trehalose and proposed that it might be a way to restrict excessive accumulation of trehalose which might do harm to plant’s metabolism. Therefore, we suggested that the ability of metabolizing trehalose might help rhizobacteria to regulate trehalose content in their surroundings. This regulation might be conducive for PGPR strains to reduce plant defense responses to root surface colonization and influence on plant-growth.

D-salicin utilization was one of the three top phenotypes which were correlated with more than three plant agronomic parameters. Salicin is an aryl β-glucoside which is found in a wide variety of plants and is used as a carbon source by many bacteria, including potential PGPR and phytopathogenic bacteria (Charaoui-Boukerzaza and Hugouvieux-Cotte-Pattat, 2013). In addition, it has been known that β-D-salicin itself don’t have anti-inflammatory or anti-proliferative activity, but can be metabolized or chemically oxidized to the pharmacological active form, salicylic acid, which is also preferred by rhizosphere bacteria (Mahdi, 2014; Hu et al., 2017; Zhalnina et al., 2018). Studies have shown that the first step of PGPR interacting with plant is to suppress or evade plant defense system for successful colonization, as same as that of plant pathogens do though the ending is so different (Lugtenberg et al., 2001; Mendes et al., 2013). One possible mechanism of PGPR impacting on the immune system of plant is to regulate immune-related signal molecules (Pieterse et al., 2012). Many studies indicate salicylic acid is one of immune-related signal molecules and plays an important role in regulating root associated microbiome (Conrath et al., 2002; Lebeis et al., 2015). In the present study, the positive correlation between metabolizing salicin and PGP ability might imply that the ability of some PGPR isolates to use salicin as carbon source or degrade it to regulate plant immune responses was crucial for their further impact on plant.

β-Methyl-D-glucoside and D-cellobiose utilizations were the other two top phenotypes and correlated with four and three parameters, respectively. β-Methyl-D-glucoside is one of main carbon sources that impact the functional diversity of the soybean rhizosphere and non-rhizosphere soil communities (Zhang et al., 2020), and also important for soil microbial construction around the roots of other legumes (Du et al., 2015). Cellobiose is a degradation product of cellulose and can act as a key carbon source to induce changes in the rhizosphere microbial composition (Shen et al., 2020). Interestingly, the degradation of cellobiose, β-methyl-D-glucoside and salicin, which are the three phenotypes most strongly correlated with PGP ability, are all connected with the activity of bacterial β-glucosidase (Faure et al., 1999; Golden and Byers, 2009; Adetunji et al., 2017). β-Glucosidases are a group of enzymes playing important roles in the processes of many plant-associated bacteria utilizing cellulose. Parasitic microorganisms may get nutrition and suppress host defenses with β-glucosidases (Voegele and Mendgen, 2011). Some previous studies have found β-glucosidases activity in root-associated strains and suggest that these enzymes may be involved in plant root colonization by plant growth-promoting bacteria (Reinholdhurek et al., 1993; Faure et al., 2001; Pathan et al., 2015). Our results also implied that β-glucosidases activity might be an important trait for PGPR strains.

Many previous studies showed that the utilization of root exudate-related nutrient substrates might be meaningful for PGPR. The results of the present study quantified the relationship between them and further suggested that the capacities of PGPR isolates to utilize these nutrient substances had the potential to replace the PGP traits tested *in vitro* as the main criteria of PGPR pre-screening. Amaya-Gomez et al. (2020) proposed a multicriteria decision analysis to evaluate PGPR strains. We agreed with their idea and suggested that the utilization of substrates could be used as one of key indicators in the evaluation system. However, we had to point out that the metabolism of nutrients by an isolate might be only a prerequisite for its action on plants, and it did not necessarily mean that the isolate could promote plant growth. Therefore, these metabolic phenotypes could only be used as “necessary but not sufficient” criteria in the preliminary screening of PGPR. In addition, the correlations between the multiple metabolic phenotypes and PGP ability of rhizobacterial isolates were demonstrated using sterilized vermiculite in the present study. It remained to be assessed whether these correlations persisted in real farmland soil. Therefore, the results of this study might not be suitable for using as a direct measure of the practical PGP ability of rhizobacterial isolates in agricultural soil environments. However, these metabolic phenotypes related with PGP ability identified in the artificially controlled environment were still of significance for the preliminary screening of PGPR, because they described the essential plant–microbe interactions with less external influences. As criteria, these metabolic phenotypes might be more widely used in a variety of soils with different environmental characteristics than the criteria obtained using a specific soil. Using these more versatile criteria that were related to basic ability of bacterial colonization in rhizosphere rather than specific PGP traits would help to retain more rhizobacterial isolates that had unknown PGP mechanisms. This was another reason why we suggested these metabolic phenotypes could be used as “necessary but not sufficient” criteria in a PGPR evaluating system.

Interestingly, significant negative correlations were observed between the utilization of one amino acid and two organic acid substrates by the rhizobacterial isolates, and their PGP ability. In addition to these three substrates, the capacities of the isolates to utilize many other amino acid and organic acid substrates were also negatively correlated with their PGP ability, albeit not significantly (Figure 4). With regard to non-pathogenic microorganisms that colonize the root or rhizosphere, most studies have mainly focused on their positive effects on plant growth and related mechanisms. Little attention has been paid to the potential negative effects of these non-pathogenic microorganisms on plants (Vessey, 2003; Backer et al., 2018). The results of the present study implied that some metabolic phenotypes of rhizobacteria might have a negative effect on plant growth. For example, low-molecular-weight organic acids are important components of root exudates and play effective roles in the dissolution of insoluble nutrients in the rhizosphere (Basak, 2019). The utilization of low-molecular-weight organic acids by rhizobacteria may change the environment of the rhizosphere niche and reduce the availability of some nutrients (Fujii et al., 2013), such as tricalcium phosphate, which is the main phosphorus source for the plants used in this study. Although the metabolism of organic acids was beneficial for bacterial colonization in the rhizosphere (Zhang et al., 2014), the present study implied that this phenotype might not be a favorable factor in promoting plant growth.

Taken together, we proposed that during preliminary PGPR screening, more attention should be paid to the metabolic phenotypes which might be related to the rhizosphere colonization ability of the isolates for colonization was the most important prerequisite for PGPR to affect plants. Although there were still many problems needed to be resolved, these phenotypes had great potential to become important indicators for preliminary soybean PGPR screening. Further, more metabolic phenotypes should be explored and evaluated based on a variety of plant–microbe interaction systems to obtain sufficient screening criteria for PGPR strains of many different taxa. Through a combination of using these metabolic criteria and more PGP traits, it could be expected that a more efficient and versatile high-throughput screening system would be established for PGPR in the future.

## Data Availability Statement

The datasets presented in this study can be found in online repositories. The names of the repository/repositories and accession number(s) can be found in the article/Supplementary Material.

## Author Contributions

PS and GW designed the experiments with equal contribution. PS and JZ performed most of the experiments, including isolation and phenotypes scanning experiments. JZ, HL, and LW performed the three inoculation experiments separately. XL, LZ, YZ, and MC analyzed the data. PS and JZ wrote the manuscript with the help of other authors. All the authors read and approved the final manuscript.

## Conflict of Interest

The authors declare that the research was conducted in the absence of any commercial or financial relationships that could be construed as a potential conflict of interest.

## Publisher’s Note

All claims expressed in this article are solely those of the authors and do not necessarily represent those of their affiliated organizations, or those of the publisher, the editors and the reviewers. Any product that may be evaluated in this article, or claim that may be made by its manufacturer, is not guaranteed or endorsed by the publisher.

## Footnotes

1
http://www.ezbiocloud.net

## Abbreviations

PGPR: plant growth-promoting rhizobacteria
IAA: indole-3-acetic acid
dpi: days post-inoculation
WCD: well color development
CI: comprehensive index
RDW: root dry weight
SDW: shoot dry weight
NDW: dry weight of per nodule
TNN: total number of nodules
RNN: number of red nodules
RN: root total nitrogen
SN: shoot total nitrogen.
