# Supplementary material for: Multiple Metabolic Phenotypes as Screening Criteria Are Correlated With the Plant Growth-Promoting Ability of Rhizobacterial Isolates
Source: Front Microbiol. 2022 Jan 5;12:747982. doi: 10.3389/fmicb.2021.747982 (PMC8767003; doi:10.3389/fmicb.2021.747982)
Supplement: Supplementary file 1 [file Table_1.docx]

Article title: Multiple metabolic phenotypes as screening criteria are correlated with the plant growth-promoting ability of rhizobacterial isolates

Journal name: Frontiers in Microbiology

Authors: Peng Shi, Jianli Zhang, Xingyue Li, Liyun Zhou, Hui Luo, Li Wang, Yafan Zhang, Minxia Chou, Gehong Wei

State Key Laboratory of Crop Stress Biology in Arid Areas, Shaanxi Key Laboratory of Agricultural and Environmental Microbiology, College of Life Sciences, Northwest A&F University, Yangling, Shaanxi 712100, PR China

Correspondence:

Peng Shi, State Key Laboratory of Crop Stress Biology in Arid Areas, Shaanxi Key Laboratory of Agricultural and Environmental Microbiology, College of Life Sciences, Northwest A&F University, Yangling, Shaanxi 712100, PR China, E-mail: shipeng27@nwafu.edu.cn, ORCID 0000-0002-1224-629X

Gehong Wei, State Key Laboratory of Crop Stress Biology in Arid Areas, Shaanxi Key Laboratory of Agricultural and Environmental Microbiology, College of Life Sciences, Northwest A&F University, Yangling, Shaanxi 712100, PR China, E-mail: weigehong@nwafu.edu.cn

**Supplementary Table 1** Rhizobacteria isolated in this study and their PGP traits

| Isolates | PGPR characteristics | | | | Number of isolates |
| --- | --- | --- | --- | --- | --- |
|  | Mineral phosphate solubilization | Siderophore production | Chitinase production | Indole acetic acid production |  |
| *Microbacterium* sp. |  |  |  |  | 1 |
| CCNWSP60 | － | － | － | ＋ |  |
| *Sphingobacterium* sp. |  |  |  |  | 5 |
| CCNWSP24-1,CCNWSP36-1, CCNWSP81-1 | － | － | － | － |  |
| CCNWSP31 | － | ＋ | － | － |  |
| CCNWSP93-2 | － | － | － | ＋ |  |
| *Bacillus* sp. |  |  |  |  | 24 |
| CCNWSP9, CCNWSP11, CCNWSP16, CCNWSP22,  CCNWSP29-1, CCNWSP34, CCNWSP35-1, CCNWSP37,  CCNWSP46, CCNWSP52, CCNWSP56, CCNWSP57,  CCNWSP64, CCNWSP69, CCNWSP77, CCNWSP79,  CCNWSP84, CCNWSP88,CCNWSP99 | － | － | ＋ | ＋ |  |
| CCNWSP2, CCNWSP76 | － | － | ＋ | － |  |
| CCNWSP14 | － | － | － | ＋ |  |
| CCNWSP75 | － | － | － | － |  |
| CCNWSP1 | － | ＋ | ＋ | ＋ |  |
| *Lysinibacillus* sp. |  |  |  |  | 9 |
| CCNWSP21, CCNWSP35, CCNWSP36, CCNWSP89,  CCNWSP90, CCNWSP91, CCNWSP96, CCNWSP102 | － | － | － | ＋ |  |
| CCNWSP63 | ＋ | － | － | ＋ |  |
| *Advenella* sp. |  |  |  |  | 2 |
| CCNWSP27-2, CCNWSP33 | ＋ | ＋ | － | ＋ |  |
| *Agrobacterium* sp. |  |  |  |  | 3 |
| CCNWSP2-1, CCNWSP26 | － | － | － | ＋ |  |
| CCNWSP100 | － | ＋ | － | ＋ |  |
| *Alcaligenes* sp. |  |  |  |  | 17 |
| CCNWSP6, CCNWSP13, CCNWSP13-2, CCNWSP13-4,  CCNWSP30, CCNWSP34-1, CCNWSP35-3, CCNWSP48,  CCNWSP60-1, CCNWSP65-1, CCNWSP76-1, CCNWSP78,  CCNWSP81, CCNWSP90-2, CCNWSP93, CCNWSP93-1 | － | ＋ | － | － |  |
| CCNWSP73-2 | － | － | － | － |  |
| *Brevundimonas* sp. |  |  |  |  | 1 |
| CCNWSP10 | － | － | － | － |  |
| *Paracoccus* sp. |  |  |  |  | 2 |
| CCNWSP6-1, CCNWSP27 | － | － | － | ＋ |  |
| *Pseudochrobactrum* sp. |  |  |  |  | 16 |
| CCNWSP4, CCNWSP11-2, CCNWSP21-1, CCNWSP23,  CCNWSP29-2, CNWSP29-3, CCNWSP35-2, CCNWSP59-3,  CCNWSP65,CCNWSP65-2, CCNWSP68, CCNWSP73,  CCNWSP81-2, CNWSP85-1,CCNWSP90-4 | － | ＋ | － | ＋ |  |
| CCNWSP25 | － | － | ＋ | ＋ |  |
| *Shinella* sp. |  |  |  |  | 1 |
| CCNWSP92 | － | － | － | ＋ |  |
| *Ensifer* (*Sinorhizobium*) sp. |  |  |  |  | 5 |
| CCNWSP17, CCNWSP19-1,CCNWSP20, CCNWSP27-1 | ＋ | － | － | ＋ |  |
| CCNWSP32 | － | － | － | ＋ |  |
| *Stenotrophomonas* sp. |  |  |  |  | 4 |
| CCNWSP15, CCNWSP47-1,CCNWSP83 | － | ＋ | ＋ | ＋ |  |
| CCNWSP72 | － | － | ＋ | ＋ |  |

＋ Positive for the trait

－ Negative for the trait
