# Supplementary material for: Multiple Metabolic Phenotypes as Screening Criteria Are Correlated With the Plant Growth-Promoting Ability of Rhizobacterial Isolates
Source: Front Microbiol. 2022 Jan 5;12:747982. doi: 10.3389/fmicb.2021.747982 (PMC8767003; doi:10.3389/fmicb.2021.747982)
Supplement: Supplementary file 4 [file Table_4.docx]

Article title: Multiple metabolic phenotypes as screening criteria are correlated with the plant growth-promoting ability of rhizobacterial isolates

Journal name: Frontiers in Microbiology

Authors: Peng Shi, Jianli Zhang, Xingyue Li, Liyun Zhou, Hui Luo, Li Wang, Yafan Zhang, Minxia Chou, Gehong Wei

State Key Laboratory of Crop Stress Biology in Arid Areas, Shaanxi Key Laboratory of Agricultural and Environmental Microbiology, College of Life Sciences, Northwest A&F University, Yangling, Shaanxi 712100, PR China

Correspondence:

Peng Shi, State Key Laboratory of Crop Stress Biology in Arid Areas, Shaanxi Key Laboratory of Agricultural and Environmental Microbiology, College of Life Sciences, Northwest A&F University, Yangling, Shaanxi 712100, PR China, E-mail: shipeng27@nwafu.edu.cn, ORCID 0000-0002-1224-629X

Gehong Wei, State Key Laboratory of Crop Stress Biology in Arid Areas, Shaanxi Key Laboratory of Agricultural and Environmental Microbiology, College of Life Sciences, Northwest A&F University, Yangling, Shaanxi 712100, PR China, E-mail: weigehong@nwafu.edu.cn

**Supplementary Table 4** The *Ratio* of the agronomic parameters of the inoculated soybean plants to the non-inoculated plants

| Treatment | Dry weight^a^ | | Total number of root nodules |
| --- | --- | --- | --- |
|  | Root | Shoot |  |
| No inoculation | 1.000±0.221 | 1.000±0.205 | 0 |
| Inoculation with CCNWSP46 | 1.618±0.317** | 1.379±0.317** | 0 |
| Inoculation with CCNWSP13-4 | 1.502±0.295** | 1.506±0.197** | 0 |
| Inoculation with CCNWSP78 | 1.462±0.185** | 1.344±0.272* | 0 |
| Inoculation with CCNWSP92 | 1.423±0.437* | 1.430±0.231** | 0 |
| Inoculation with CCNWSP15 | 1.519±0.289** | 1.514±0.406** | 0 |
| Inoculation with CCNWSP60 | 1.381±0.311* | 1.389±0.292** | 0 |
| Inoculation with CCNWSP31 | 1.311±0.370 | 1.308±0.233 | 0 |
| Inoculation with CCNWSP26 | 1.425±0.309** | 1.185±0.298 | 0 |
| Inoculation with CCNWSP76 | 1.261±0.247 | 1.144±0.238 | 0 |
| Inoculation with CCNWSP2 | 1.104±0.244 | 1.102±0.228 | 0 |
| Inoculation with CCNWSP68 | 1.267±0.447 | 1.254±0.281 | 0 |
| Inoculation with CCNWSP10 | 0.882±0.227 | 1.090±0.247 | 0 |
| Inoculation with CCNWSP11 | 1.279±0.299 | 1.176±0.242 | 0 |
| Inoculation with CCNWSP27 | 1.015±0.123 | 1.169±0.273 | 0 |
| Inoculation with CCNWSP13-2 | 1.086±0.346 | 1.053±0.264 | 0 |
| Inoculation with CCNWSP30 | 1.169±0.292 | 1.132±0.196 | 0 |
| Inoculation with CCNWSP21 | 1.231±0.278 | 1.252±0.443 | 0 |
| Inoculation with CCNWSP4 | 1.077±0.202 | 1.154±0.257 | 0 |
| Inoculation with CCNWSP33 | 0.975±0.316 | 1.010±0.320 | 0 |
| Inoculation with CCNWSP21-1 | 1.178±0.341 | 1.162±0.250 | 0 |
| Inoculation with CCNWSP25 | 1.117±0.507 | 1.012±0.273 | 0 |

^a^ The data are presented as means ± standard deviations (*n* = 9 for the inoculation treatments, *n* = 24 for the non-inoculation treatment).

*, Significant different at *p* < 0.05 and **, significant different at *p* < 0.01, compared to the data of the non-inoculation treatment according to Dunnett’s test.
