# Supplementary material for: Multiple Metabolic Phenotypes as Screening Criteria Are Correlated With the Plant Growth-Promoting Ability of Rhizobacterial Isolates
Source: Front Microbiol. 2022 Jan 5;12:747982. doi: 10.3389/fmicb.2021.747982 (PMC8767003; doi:10.3389/fmicb.2021.747982)
Supplement: Supplementary file 6 [file Table_6.docx]

Article title: Multiple metabolic phenotypes as screening criteria are correlated with the plant growth-promoting ability of rhizobacterial isolates

Journal name: Frontiers in Microbiology

Authors: Peng Shi, Jianli Zhang, Xingyue Li, Liyun Zhou, Hui Luo, Li Wang, Yafan Zhang, Minxia Chou, Gehong Wei

State Key Laboratory of Crop Stress Biology in Arid Areas, Shaanxi Key Laboratory of Agricultural and Environmental Microbiology, College of Life Sciences, Northwest A&F University, Yangling, Shaanxi 712100, PR China

Correspondence:

Peng Shi, State Key Laboratory of Crop Stress Biology in Arid Areas, Shaanxi Key Laboratory of Agricultural and Environmental Microbiology, College of Life Sciences, Northwest A&F University, Yangling, Shaanxi 712100, PR China, E-mail: shipeng27@nwafu.edu.cn, ORCID 0000-0002-1224-629X

Gehong Wei, State Key Laboratory of Crop Stress Biology in Arid Areas, Shaanxi Key Laboratory of Agricultural and Environmental Microbiology, College of Life Sciences, Northwest A&F University, Yangling, Shaanxi 712100, PR China, E-mail: weigehong@nwafu.edu.cn

**Supplementary Table 6** The *Ratio* of the agronomic parameters of soybean plants co-inoculated with rhizobacteria and *Sinorhizobium* sp. CCNWSX1528 to those inoculated with *Sinorhizobium* sp. CCNWSX1528 alone

| Treatment | Dry weight | | | Number of nodules | | Total nitrogen | | Comprehensive index |
| --- | --- | --- | --- | --- | --- | --- | --- | --- |
|  | Root | Shoot | Per nodule | Total nodules | Red nodules | Root | Shoot |  |
| CCNWSX1528 alone | 1.000±0.081 | 1.000±0.088 | 1.000±0.147 | 1.000±0.077 | 1.000±0.154 | 1.000±0.080 | 1.000±0.124 | 1.000 |
| CCNWSX1528 + CCNWSP46 | 1.631±0.243** | 1.295±0.153** | 1.397±0.446** | 1.582±0.164** | 1.922±0.237** | 1.560±0.205** | 1.212±0.104** | 1.514 |
| CCNWSX1528 + CCNWSP13-4 | 1.451±0.125** | 1.191±0.076** | 1.222±0.126* | 1.661±0.139** | 1.970±0.470** | 1.369±0.140** | 1.262±0.260** | 1.447 |
| CCNWSX1528 + CCNWSP78 | 1.513±0.206** | 1.123±0.088 | 1.182±0.196 | 1.496±0.200** | 1.385±0.302** | 1.401±0.248** | 1.173±0.118* | 1.325 |
| CCNWSX1528 + CCNWSP92 | 1.327±0.240** | 1.182±0.123** | 1.134±0.119 | 1.444±0.152** | 1.704±0.194** | 1.248±0.205** | 1.168±0.165* | 1.315 |
| CCNWSX1528 + CCNWSP15 | 1.386±0.191** | 1.155±0.156** | 1.128±0.114 | 1.353±0.147** | 1.553±0.218** | 1.387±0.208** | 1.224±0.132** | 1.312 |
| CCNWSX1528 + CCNWSP60 | 1.333±0.116** | 1.201±0.061** | 1.248±0.239* | 1.241±0.136** | 1.350±0.240** | 1.314±0.146** | 1.154±0.059 | 1.263 |
| CCNWSX1528 + CCNWSP31 | 1.232±0.216** | 1.202±0.138** | 1.326±0.099** | 1.206±0.132* | 1.333±0.183** | 1.278±0.145** | 1.116±0.133 | 1.242 |
| CCNWSX1528 + CCNWSP26 | 1.313±0.159** | 1.215±0.141** | 1.313±0.135** | 1.158±0.104 | 1.184±0.116 | 1.323±0.137** | 1.150±0.188 | 1.237 |
| CCNWSX1528 + CCNWSP76 | 1.199±0.077* | 1.059±0.071 | 1.019±0.118 | 1.336±0.127** | 1.225±0.176 | 1.185±0.074* | 1.098±0.143 | 1.160 |
| CCNWSX1528 + CCNWSP2 | 1.046±0.125 | 1.094±0.124 | 1.335±0.189** | 1.068±0.121 | 1.169±0.183 | 1.103±0.132 | 1.085±0.126 | 1.129 |
| CCNWSX1528 + CCNWSP68 | 1.167±0.235 | 1.044±0.101 | 1.094±0.180 | 1.086±0.258 | 1.104±0.154 | 1.192±0.270* | 1.200±0.158** | 1.127 |
| CCNWSX1528 + CCNWSP10 | 1.193±0.136* | 1.038±0.051 | 1.136±0.198 | 1.095±0.209 | 1.226±0.295 | 1.125±0.129 | 0.994±0.106 | 1.115 |
| CCNWSX1528 + CCNWSP11 | 1.078±0.117 | 1.012±0.096 | 1.084±0.171 | 1.108±0.156 | 1.280±0.248 | 1.096±0.126 | 1.096±0.122 | 1.108 |
| CCNWSX1528 + CCNWSP27 | 0.937±0.110 | 1.012±0.055 | 1.019±0.106 | 1.120±0.151 | 1.295±0.187* | 0.881±0.142 | 1.062±0.063 | 1.047 |
| CCNWSX1528 + CCNWSP13-2 | 1.010±0.111 | 0.936±0.109 | 0.909±0.118 | 1.051±0.208 | 1.202±0.314 | 1.008±0.104 | 0.999±0.140 | 1.016 |
| CCNWSX1528 + CCNWSP30 | 1.060±0.199 | 0.972±0.097 | 0.986±0.171 | 1.020±0.086 | 1.063±0.179 | 1.008±0.160 | 0.974±0.094 | 1.012 |
| CCNWSX1528 + CCNWSP21 | 1.093±0.143 | 1.005±0.183 | 1.209±0.204 | 0.770±0.170* | 0.890±0.135 | 1.109±0.095 | 1.007±0.150 | 1.012 |
| CCNWSX1528 + CCNWSP4 | 1.000±0.118 | 0.977±0.055 | 1.134±0.143 | 0.973±0.086 | 1.082±0.208 | 0.933±0.092 | 0.958±0.145 | 1.008 |
| CCNWSX1528 + CCNWSP33 | 1.142±0.162 | 0.911±0.140 | 0.908±0.229 | 0.878±0.194 | 0.872±0.327 | 1.094±0.104 | 0.999±0.150 | 0.972 |
| CCNWSX1528 + CCNWSP21-1 | 0.984±0.126 | 0.939±0.083 | 0.907±0.187 | 0.924±0.181 | 0.950±0.119 | 0.994±0.088 | 0.966±0.118 | 0.952 |
| CCNWSX1528 + CCNWSP25 | 0.917±0.225 | 0.869±0.158 | 1.016±0.206 | 0.822±0.377 | 0.990±0.389 | 1.002±0.176 | 0.945±0.127 | 0.937 |

The data are expressed as means ± standard deviations (*n* = 9 for the co-inoculation treatments, *n* = 24 for the single inoculation treatment).

*, Significant different at *p* < 0.05 and **, significant different at *p* < 0.01, compared to the data of single inoculation according to Dunnett’s test.
